# Democratizing the spatial view: STAMP technology from an analytical perspective

**DOI:** 10.70401/EXO.2026.0012

**Published:** 2026-06-03

**Authors:** Suresh Poudel, Felipe Segato Dezem, Luciano G. Martelotto, Jasmine T. Plummer, Douglas R. Green

**Affiliations:** 1 Department of Immunology, St. Jude Children’s Research Hospital, Memphis, TN 38105, USA.; 2 Center for Spatial Omics, St. Jude Children’s Research Hospital, Memphis, TN 38105, USA.; 3 Waters Biosciences (Formerly BD Biosciences), Milpitas, CA 95035, USA.

**Keywords:** STAMP, single-cell transcriptomics, multimodal imaging, cell morphology, spatial biology, morpho-transcriptomic, deep generative models, gene program scoring

## Abstract

Single-cell RNA sequencing (scRNA-seq) has transformed the profiling of cellular heterogeneity; however, tissue dissociation for scRNA-seq eliminates three critical classes of information that often define cell state: spatial organization, morphology, and protein localization. Spatial transcriptomics partially restores this context, yet many platforms are limited by cost, throughput, or experimental complexity. Single-cell transcriptomics analysis and multimodal profiling (STAMP) tackle these limitations by immobilizing cells or nuclei on a slide, enabling high-content imaging prior to molecular readout and consequently preserving each cell’s visual characteristics alongside high-plex transcript and/or protein measurements. This mini review focuses on the downstream analytical workflow for STAMP datasets using Python, illustrating how standard single-cell methods can be applied to image-derived cell-by-feature matrices enriched with per-cell covariates. The pooled MIX sub-STAMP serves as a working example to illustrate practical steps for quality control, normalization, dimensionality reduction, clustering or label transfer, and program-level interpretation. Morpho-transcriptomic coupling is highlighted as an exploratory strategy that links inferred gene programs to image-derived morphology. Collectively, these analyses establish STAMP as a practical bridge between single-cell transcriptomics and quantitative cell phenotype, enabling interpretable state inference that can be validated by imaging-derived measurements.

## 1. Introduction

Over the past decade, single-cell genomics has required a trade-off between molecular-level depth and preservation of phenotypic context. Droplet-based single-cell RNA sequencing (scRNA-seq) enables highly scalable gene expression profiling, but this approach requires tissue dissociation, which can lead to loss of cellular shape, polarity, and neighborhood relationships^[1]^. In many biological systems, these non-molecular properties are central indicators of function and state. Consequently, the loss of morphology and local context complicates data interpretation and may obscure distinctions among genuine biological variation and technical variations introduced during dissociation.

Spatial transcriptomics has been developed to recover contextual information by measuring gene expression in situ^[2,3]^. However, common approaches face practical limitations. Some platforms aggregate signals from multiple cells per spot, while higher-resolution methods are often costly, slower, or operationally complex, restricting scalability^[4]^. This necessitates a method that maintains the throughput and modularity of dissociation-based workflows while restoring the phenotypic richness provided by microscopy. Spatial omics assays can be categorized by the site of measurement (intact tissue versus dissociated cells) and by the definition of “spatial” information (native tissue geography versus cell-intrinsic phenotypes such as morphology, polarity, and marker localization).

Single-cell transcriptomics analysis and multimodal profiling (STAMP) provides a pragmatic, cost-effective solution by pausing the single-cell workflow after dissociation but before lysis. By immobilizing dissociated cells or nuclei onto a slide, STAMP preserves coordinate-registered phenotypic information, including cell size, shape, and optional immunofluorescence markers, before RNA and/or protein measurements are obtained from the same immobilized cells^[5]^. Within this system, STAMP is classified as a dissociation-based slide-array method that retains cell-intrinsic spatial phenotype while sacrificing native tissue coordinates, therewith positioning it between droplet scRNA-seq and intact-section spatial methods (Table 1).

Analytically, this approach converts a conventional count-matrix experiment into an image-linked single-cell dataset, thereby retaining morphology and geometry as primary covariates alongside gene expression. The downstream analytical consequence of this design is that, once STAMP outputs are formatted as an AnnData-style cell-by-feature matrix with aligned per-cell metadata, they become amenable to analysis using the modern Python single-cell ecosystem.

We use Scanpy for preprocessing, visualization, and graph-based analyses (normalization, neighborhood construction, Uniform Manifold Approximation and Projection (UMAP), and Leiden community detection for unsupervised clustering, and differential expression)^[12–14]^, and deep generative latent modeling with single-cell variational inference tools (scVI) as an integration backbone^[15]^. We then apply single-cell ANnotation using variational inference (scANVI) for semi-supervised projection of query cells onto labeled references while producing calibrated posterior probabilities for each assignment^[15–17]^.

This mini review illustrates one representative workflow using Scanpy, scVI, and scANVI. Other methods (e.g., alternative integration approaches such as Harmony or batch balanced k nearest neighbours (BBKNN), and trajectory inference methods such as diffusion pseudotime) are conceptually compatible with STAMP data but are not demonstrated here.

Collectively, this workflow enables strict quality control, uncertainty-aware label transfer, and state interpretation, all of which can be validated directly against image-derived phenotypes. This interpretability loop is challenging to achieve with sequencing-only assays.

This article presents an analytical perspective on STAMP downstream analysis, providing a practice-oriented perspective that synthesizes workflow considerations and uses one published dataset as an illustrative example rather than a comprehensive benchmark. This fills a gap between technical publications describing the STAMP methodology and user-level guidance for data analysis in Python.

The following sections provide (i) an end-to-end summary of the downstream pipeline, (ii) specific guidance for image-aware quality control, and (iii) examples of validating computational inferences against measured morphology. STAMP integrates per-cell morphology (and optional protein localization) with gene expression in the same cells, permitting direct testing of transcriptional programs against measured phenotypes. Descriptions of the analytical tools used in this mini review are summarized in Table 2.

### End-to-end downstream pipeline summary:

1. Image-derived matrix construction: export cell × gene (and optional protein) matrices with per-cell coordinates, segmentation IDs, and morphology features.
2. Image-aware quality control (QC): filter obvious artifacts using morphology (e.g., area/shape) and negative-control probes in addition to RNA counts/complexity.
3. Normalization and feature selection: library-size normalization/log1p and selection of informative genes for embeddings.
4. Latent modeling and integration: principal component analysis (PCA)/UMAP for exploratory analysis, or scVI for batch-aware latent integration.
5. Label transfer with uncertainty: scANVI projection onto labeled references and retention of posterior probabilities for review/abstention.
6. Composition checks: global and per-field-of-view mixture proportions as a diagnostic for technical drift.
7. Program scoring: module scoring for curated pathways and differential testing as needed.
8. Morpho-transcriptomic coupling: test inferred states/programs against morphology (e.g., area, aspect ratio) and back-project results to images.

We recommend storing STAMP outputs in an AnnData object with layers[“counts”] for raw counts, X or layers for normalized matrices, obs for per-cell metadata (including morphology and QC flags), obsm for embeddings (e.g., X_umap, X_scanvi), and uns/obsp for parameters and neighbor graphs.

#### Normalization considerations:

STAMP produces two distinct data types, i.e., gene expression matrices and image-derived intensity features (fluorescence-derived when available), each with different statistical properties. Gene expression matrices typically undergo library-size normalization (e.g., counts per 10,000, log1p transformation), as in standard scRNA-seq. In contrast, intensity features can be affected by technical biases (e.g., illumination gradients, autofluorescence) and may require background subtraction, field-of-view–level intensity scaling, and/or robust z-scoring. Users should account for these differences when integrating morphological features with gene expression.

## 2. Workflow (Computational Architecture from an Analytical Perspective)

**Figure 1.**
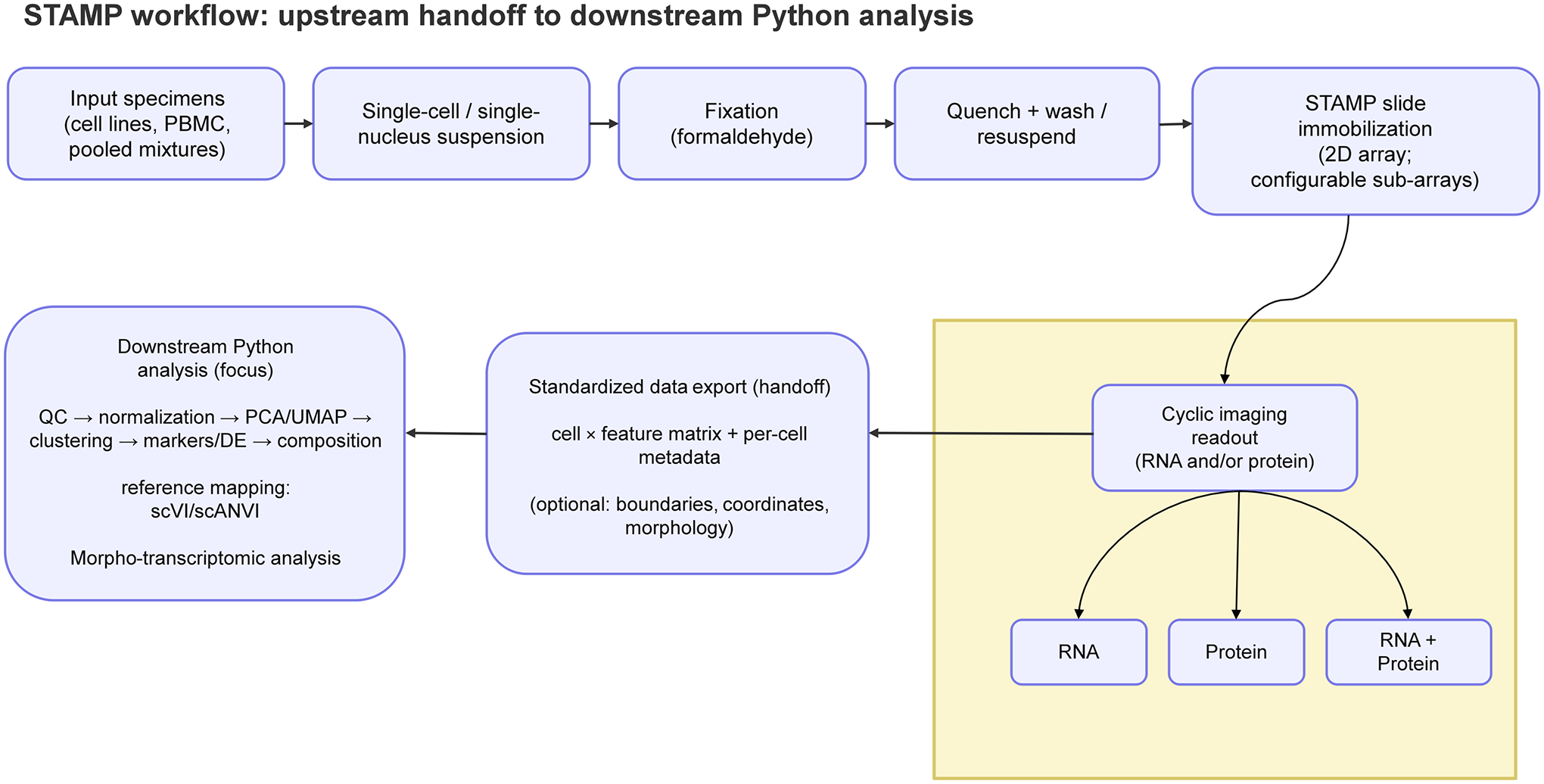
STAMP workflow and downstream analysis. Dissociated cells or nuclei are immobilized on a slide, imaged (RNA +/− protein), and exported as an analysis-ready cell × feature matrix linked to per-cell morphology and coordinates. Downstream Python analysis applies standard single-cell steps (QC, normalization, embedding/latent modeling, clustering/label transfer, morpho-transcriptomic analysis, and interpretation), with results traceable back to the images. STAMP: single-cell transcriptomics analysis and multimodal profiling; QC: quality control; PBMC: peripheral blood mononuclear cell; PCA: principal component analysis; UMAP: Uniform Manifold Approximation and Projection; DE: differential expression; scVI: single-cell variational inference; scANVI: single-cell ANnotation using variational inference.

STAMP begins with the preparation of a single-cell or single-nucleus suspension (e.g., dissociated tissue cells, peripheral blood mononuclear cells (PBMCs), or isolated nuclei). These suspensions are immobilized (“stamped”) onto a glass slide. After immobilization, cyclic imaging enables high-plex readout of RNA and/or protein at subcellular resolution, and recent benchmarking studies contextualize platform performance in formalin-fixed paraffin-embedded (FFPE) tissues (Figure 1)^[9,18]^.

The analysis of STAMP data can be understood as a two-layer pipeline that cleanly separates platform-specific image processing from platform-agnostic single-cell inference, as demonstrated in Figure 1.

The upstream workflow begins with the primary imaging outputs produced by the instrument and/or vendor software. Its goal is to construct a faithful per-cell data model in a shared coordinate system: corrected images, cell masks or polygons^[19,20]^, per-molecule detections with spatial coordinates, and per-cell feature matrices for RNA and/or proteins. During this stage, metadata associated with multimodal data, such as cell identifiers, must remain consistent across segmentation, molecular tables, images, and coordinate frames and be maintained. This is ultimately used in any downstream computation and can be mapped back to the microscope evidence.

Since STAMP retains images and geometry, upstream QC is image-first. Artifacts such as border effects (artifacts arising from cells at the edges of imaging fields of view, where illumination non-uniformity and processing artifacts are more common), clumping, or segmentation failures can be visually flagged and filtered using morphometric features (e.g., cell area) rather than inferred solely from expression. The key output of the upstream stage is therefore not simply a matrix, but an analysis-ready object in which each cell contains counts, coordinates, boundaries, and phenotype-linked metadata.

Here, we have focused on the downstream workflow, demonstrating how Python, as the analytical language, can be used to transform image-derived count matrices (RNA) into biological insights.

The downstream workflow follows the logic of scRNA-seq analysis (filtering, normalization, integration, dimensionality reduction, neighborhood graph construction, and clustering) in an image-native setting, where morphology and geometry can serve as covariates, stratifiers, or diagnostic signals. Standard transformations (library-size normalization and log scaling) support embeddings such as PCA/UMAP and network-based clustering, while multimodal annotation can incorporate both molecular features and image-derived phenotypes.

Additionally, STAMP can utilize cluster labels, gene programs, and inferred states, and project them back onto cell boundaries and images to confirm how computational groupings relate to visual phenotypes. With these components in place, downstream analyses support tasks such as marker identification, differential testing, gene program scoring, and trajectory inference whenever the experimental design warrants them. In this sense, STAMP democratizes multimodal single-cell analysis by providing a computational system that links phenotype to genotype across aligned modalities rather than relying on an implicit assumption.

### 2.1 Downstream analysis: Reference mapping and marker validation of a pooled mixture

To illustrate what a typical STAMP downstream workflow looks like in practice, we re-analyzed a published pooled “MIX” sub-STAMP from a multi-sample slide array (Figure 1^[5]^), where three cancer cell lines were profiled in a 1:1:1 mixture.

**Figure 2.**
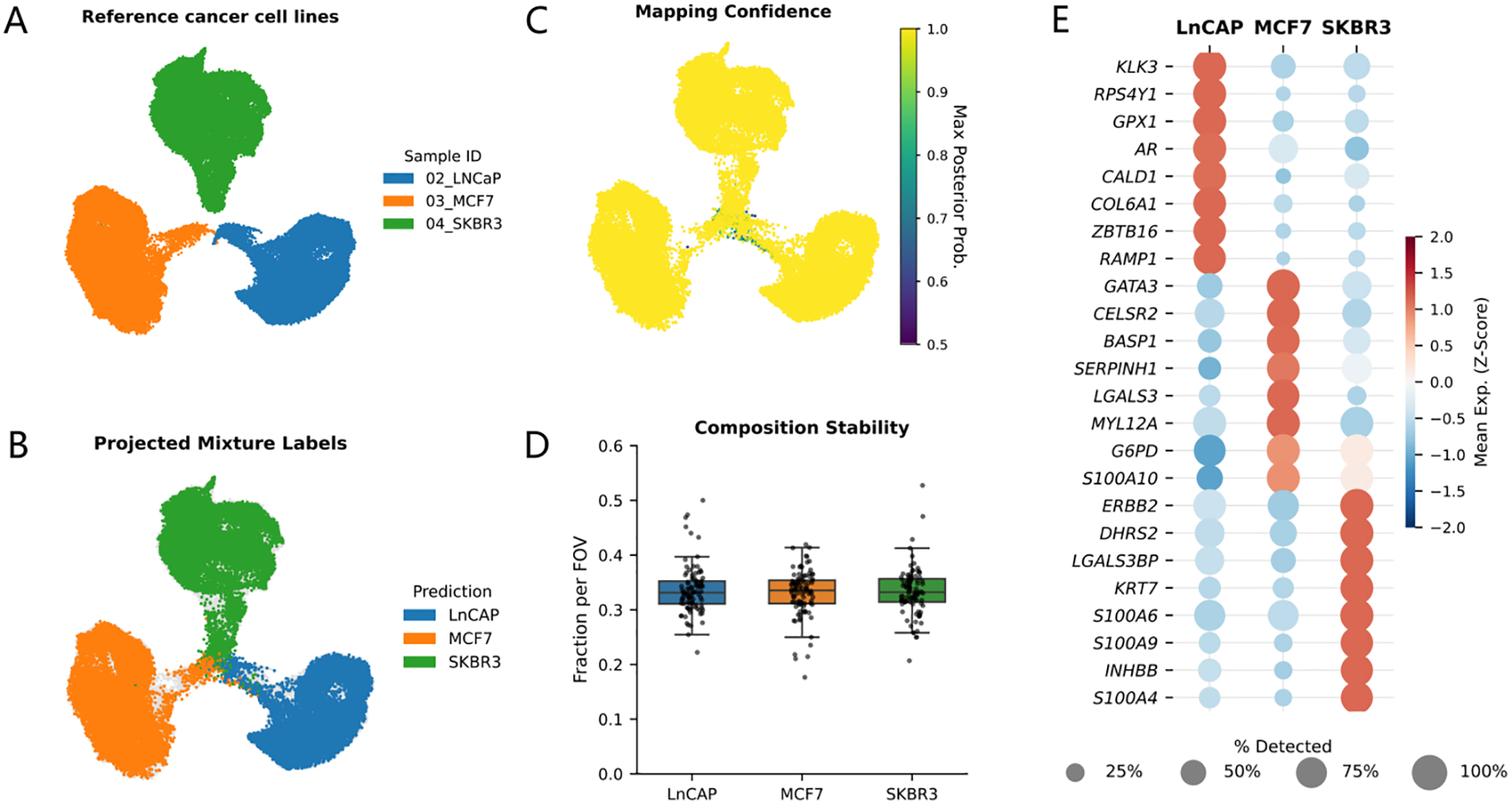
Reference mapping and validation readouts for the STAMP pooled-mixture slide using scANVI. (A) Reference embedding of pure cell lines; (B) MIX cells projected and colored by predicted identity; (C) Mapping confidence (max posterior probability); (D) Global and per-field-of-view mixture composition as a QC check; (E) Marker expression summary supports predicted labels. STAMP: single-cell transcriptomics analysis and multimodal profiling; scANVI: single-cell ANnotation using variational inference; QC: quality control.

Analytically, this design is ideal for a method-focused demonstration because it provides an internal reference. The pure sub-STAMPs define high-confidence molecular “anchors,” and the pooled MIX acts as an unlabeled query that should resolve into the same latent structure (Figure 2A,B).

Our downstream objective is therefore not the discovery of new biology, but the verification of computational fidelity, i.e., whether a standard single-cell pipeline, adapted to STAMP’s imaging-derived count matrix, can recover the expected composition, detect mapping uncertainty, and summarize differentiating features.

This mini review uses the MIX cell line mixture as an illustrative example. The workflow principles described here apply to more complex biological scenarios including tumor microenvironments, perturbation studies, and time-series analyses, though these applications are not demonstrated here.

### 2.2 Technical validation: Benchmarking the pipeline with the MIX slide

We filtered cells in two stages. First, transcriptomic QC removed cells below count or gene-detection thresholds or outside the accepted area range. Second, before morpho-transcriptomic analysis, a morphology-based filter excluded cells with low transcript content, abnormal size or shape, or high background signal. QC plots are in Figure S1.

A key decision in these STAMP samples for downstream analysis is how to treat the MIX sample relative to the pure references. To address this, we first utilized de novo PCA/Leiden clustering as an unsupervised sanity check on the same QC-filtered four-sample dataset. Leiden broadly recovered the major cell-line structure and distributed MIX cells across the expected identity-enriched regions, although a small ambiguous fraction (3.4%) remained unresolved as illustrated in Figure S2. The average distribution of the MIX population with respect to field-of-views (FOVs) ranges from (31.4% to 33.0%) as shown in Figure S2D. With this data, we performed differential expression (DE) analysis. Top 8 markers for each resolved MIX population are shown in Figure S2E. We then used a semi-supervised reference mapping strategy (scVI→scANVI) as the primary framework for downstream analysis. Instead of clustering everything de novo and then attempting to reconcile cluster identities post hoc, we use a semi-supervised reference mapping strategy (scVI→scANVI).

Conceptually, the pure sub-STAMPs provide labeled examples (cell line identities), while the MIX cells are treated as “unknown” during training and then projected into the shared latent space. This approach has two benefits that are particularly relevant to imaging-based assays. First, it isolates biological identity from technical structure by clearly modeling latent variables. Second, it provides a calibrated per-cell prediction score (maximum posterior probability), which is essential when image-based count matrices may contain low-content or ambiguous cells.

The resulting latent embedding functions as the backbone for all downstream summaries. In the combined UMAP, the MIX cells, when colored by scANVI-predicted identity, resolve cleanly into the same regions as defined by the reference cell lines (Figure 2A,B). This indicates that the mixture is largely explained by the reference classes rather than by batch-specific artifacts.

Importantly, the mapping step produces continuous confidence scores; plotting this confidence on the same embedding provides a useful diagnostic (Figure 2C). High-confidence assignments typically populate the interiors of reference regions, whereas lower-confidence cells concentrate along boundaries or low-signal neighborhoods. In the STAMP setting, such uncertainty may reflect true intermediate states, technical sparsity, segmentation/decoding artifacts, or damaged cells; the confidence score provides an analytic handle to distinguish “cells that don’t fit” from genuine biological heterogeneity.

We further inspected the low confidence scANVI assignments and found they were rare. Only 155/162,881 cells (0.095%) had scanvi_maxprob < 0.8, and only 6/162,881 (0.004%) fell below 0.5 (treated as unassigned), indicating that uncertainty-driven cases comprised a very small fraction of the dataset.

After label projection, we summarize the MIX composition at two levels. The first is global composition; the fraction of MIX cells assigned to each reference class should approximate the known mixture ratio and serve as an immediate sanity check (Figure 2D). While a perfect 1:1:1 recovery is not expected (due to differences in capture efficiency, viability, or panel sensitivity), the overall proportions serve as a major checkpoint that the downstream pipeline is behaving sensibly.

The second is local composition stability. When FOV annotations are available, composition can be computed per FOV and visualized as a distribution (Figure 2D) with MIX population ranging from 32.7% to 34.1%. This step detects spatially structured technical variation introduced by imaging cycles, illumination, bead density, or local cell density. In a robust experiment, FOV-to-FOV composition fluctuates narrowly around the global mean; large deviations may signal localized quality issues warranting upstream inspection.

### 2.3 Biological interpretation: Marker and program readouts

With a stable latent space and consistent composition established, the next downstream layer is interpretability. To identify features that explain why the mapped identities separate, we performed DE analysis on the scANVI-predicted groups and visualized the results as a compact marker dot plot (Figure 2E). Although the labels are computationally assigned and the panel is targeted, the recovery of canonical lineage programs serves as a strong internal consistency test, confirming that the image-derived count matrix is sufficiently coherent for standard single-cell statistics.

This visualization, where dot size represents detection rate and color indicates mean expression, is highly valuable for STAMP data because it explicitly displays sparsity. By distinguishing genes that are consistently detected (robust markers) from those driven by high counts in rare cells, the dot plot bridges the gap between statistical separation and measurement stability, ensuring that defined clusters rely on reproducible imaging signals rather than technical artifacts.

Overall, Figure 2 highlights a practical template for STAMP analysis: integrate pure and mixture samples in a shared latent model (Figure 2A,B), project unlabeled cells with scANVI, validate predictions using confidence and composition stability (Figure 2C,D), and summarize identity with marker statistics and compact expression visualizations (Figure 2E).

DE analysis recovers canonical lineage markers that validate cell identity: KLK3 (PSA) and AR for the prostate line LNCaP, GATA3 for the luminal breast line MCF7, and ERBB2 (HER2) for the HER2-amplified line SKBR3^[21,22]^ (Figure 2E). Additionally, the detection of the Y-linked gene RPS4Y1 in LNCaP cells but not in the female breast cancer lines (Figure 2E) confirms the male origin of this cell line.

In the broader context of the STAMP study, where experimental conditions exist (e.g., perturbations, time points, or treatments), the same framework applies naturally. Labels can represent conditions or cell states; confidence can be used to gate ambiguous cells, and DE can be performed either within mapped identities or across conditions within each identity. The downstream ecosystem of STAMP data analysis closely mirrors modern scRNA-seq practice while preserving the crucial advantage that each cell’s molecular profile remains directly linked back to its imaging-derived phenotype.

### 2.4 Gene module scoring

We constructed simple program “signatures” as small gene sets drawn from commonly used markers for each pathway/state as follows: androgen response (KLK3, AR, FKBP5, TMPRSS2, NKX3–1), estrogen response (ESR1, PGR, GATA3, FOXA1, TFF1, GREB1), HER2 signaling (ERBB2, GRB7, STARD3, PGAP3, CDH1), and G2/M (MKI67, TOP2A, CDK1, CCNB1, CENPF, AURKA). Because STAMP uses targeted panels, not all signature genes were present. Scores were therefore based only on the genes available in the panel: androgen response 3/5 (KLK3, AR, FKBP5), estrogen response 3/6 (ESR1, PGR, GATA3), HER2 signaling 2/5 (ERBB2, CDH1), and G2/M 3/6 (MKI67, TOP2A, CENPF). These program scores should be interpreted as partial-coverage proxies rather than full pathway reconstructions.

Program scores were computed using Scanpy’s tl.score_genes, which estimates a signature score as the mean expression of signature genes relative to expression-matched control genes. While this approach is standard in scRNA-seq, targeted panels reduce the available universe of control genes, potentially increasing sensitivity to panel composition and scoring hyperparameters (e.g., binning and control gene counts). Because the STAMP 1K panel still provides sufficient background genes and our use case is comparative (within-dataset) visualization, we used score_genes for interpretability.

As an alternative, targeted panels can also support control-free scoring (e.g., z-scoring each gene across cells, then averaging within a signature) or rank-based enrichment methods (e.g., AUCell)^[23]^, which may be preferable when the control pool is limited.

### 2.5 Morphology as an analysis variable, not an illustration

A distinctive analytical advantage of STAMP is that morphology becomes a measurable covariate rather than a qualitative annotation. Cell shape, size, and structural polarization can be quantified directly from the same images used for molecular decoding.

In practice, classical image features such as area, eccentricity, perimeter, nuclear-to-cytoplasmic ratios (when available), texture descriptors, and marker localization^[24]^, can be stored alongside the expression matrix and treated as first-class predictors or stratification variables. Rather than asking whether a transcriptional program implies migration, activation, or stress, STAMP supports analyses that explicitly test whether visual phenotypes co-vary with gene programs across large numbers of cells.

### 2.6 Resolving transcriptional ambiguity with image-aware QC

STAMP is a practical arbiter when transcriptomics alone is ambiguous, especially at the boundary between biology and artifact. Dissociation-based assays frequently exhibit generic stress-like signatures and coverage losses that are difficult to interpret when the only evidence is a count matrix^[25–27]^. In STAMP, the imaging layer provides orthogonal validation: segmentation integrity, gross morphology, and border proximity, which could be used to flag damaged cells, debris, merged objects, or edge artifacts before their shape embeddings and clusters are generated. Moreover, some apparent doublets may arise from segmentation-driven multiplets and can be reduced by re-segmentation or boundary refinement rather than blanket exclusion.

Although we did not apply segmentation-free (transcript-guided) cell delineation approaches in this re-analysis, such methods are conceptually compatible with STAMP and may further improve robustness in challenging samples. Together, this shifts QC from a purely statistical filtering task to a multimodal screening step, where expression thresholds are complemented by morphology-derived gates. Concretely, in the 1K-gene STAMP MIX dataset (162,881 cells), we flagged 4.44% of cells by image-aware QC using simple, reportable gates. Debris/low-content cells were defined as Area.um2 < 95.7 μm^2^ and/or low RNA complexity (n_counts < 100 or n_genes_by_counts < 30; 0.99% flagged); merged/doublet-like objects as Area.um2 > 628.5 μm^2^ (1.000%); segmentation shape outliers as AspectRatio > 2.47 (99.5th percentile; 0.495%); and high background/decoding noise as negprobe_frac > 0.05 or falsecode_frac > 0.02 (2.08%). These results are shown in Figure S1.

Full QC visualizations are not included in this mini review; however, we recommend generating pre- and post-filtering distributions for morphometric and molecular features, alongside visual inspection of flagged cells in image space, for any real-world STAMP analysis. Segmentation quality can be assessed qualitatively using platform-specific visualization tools (e.g., AtoMx for CosMx, Xenium Explorer for Xenium) and quantitatively using re-segmentation approaches when necessary (e.g., fastReseg, Proseg).

### 2.7 Morpho-transcriptomics as a downstream analysis paradigm

A central advantage of STAMP is that each segmented cell is represented as a unified record that links (i) a cell × gene expression vector with (ii) image-derived morphology and intensity features (e.g., area, aspect ratio, marker-channel means/maxima, and spatial coordinates)^[5]^. In practice, this makes downstream analysis “multi-axis” by default; expression-based embeddings and labels can be directly compared to morphometric distributions without requiring post hoc alignment between imaging and sequencing outputs.

In downstream Python analysis^[12]^, morphology can play three complementary roles. First, it can serve as an outcome variable to test whether transcriptional states predict phenotypes such as cell size or shape. Second, it can act as a covariate/confounder, enabling analyses that control for size, segmentation artifacts, or stain intensity when comparing gene programs across identities. Third, it provides an orthogonal annotation axis, allowing transcriptionally defined clusters to be subdivided into morphologically distinct subclusters and enabling quality checks by mapping outliers back to the physical tissue coordinates.

Collectively, these operations support an iterative interpretability cycle (quantify→infer→validate→refine) in which image-derived features are treated as evidence that can confirm or challenge purely transcript-based conclusions.

### 2.8 From markers to mechanisms: Program-level inference and morpho-transcriptomic coupling

**Figure 3.**
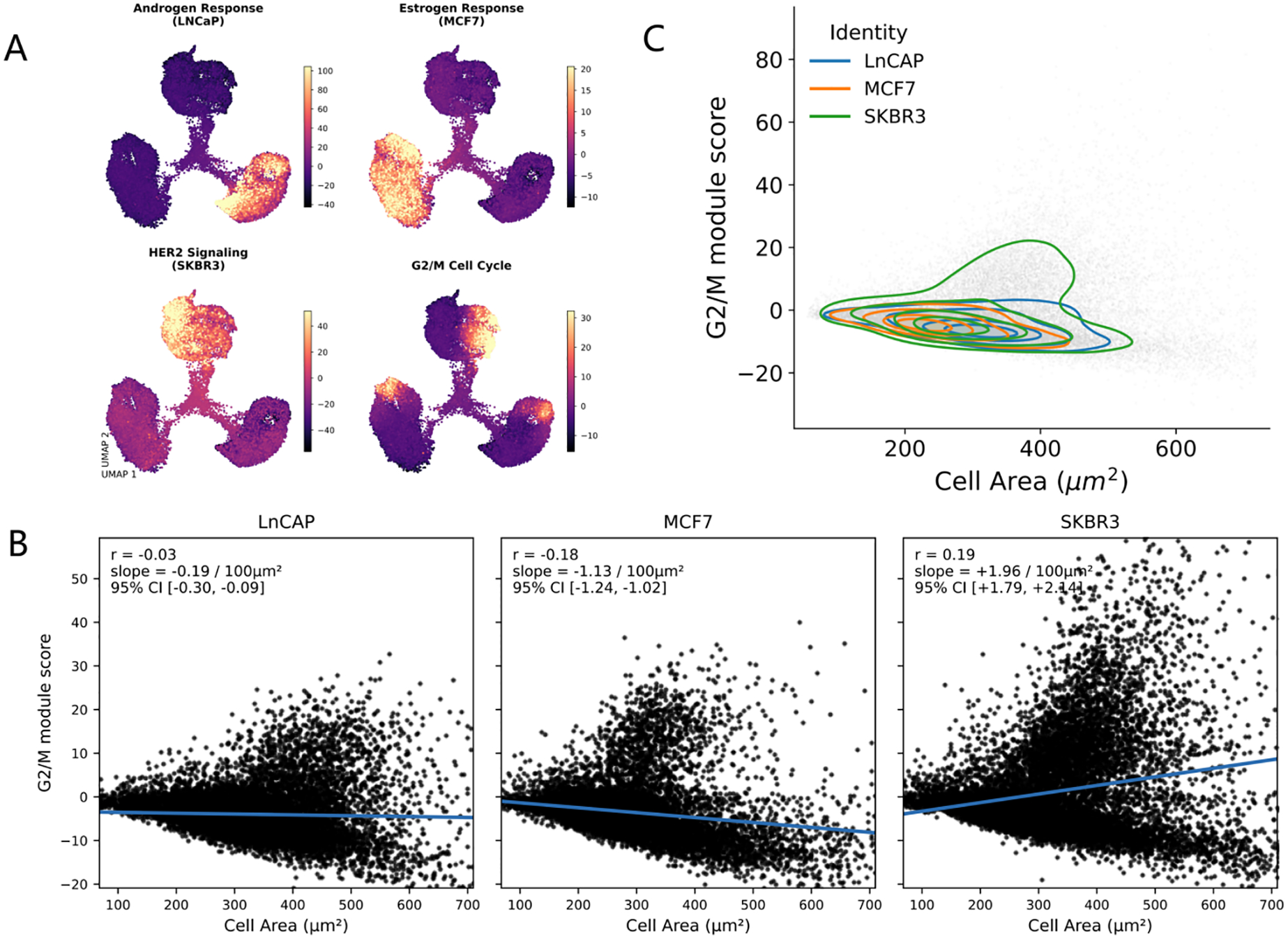
Program scoring and morpho-transcriptomic coupling in STAMP MIX. (A) Module scores for curated programs projected onto the embedding; (B) Relationship between cell area (μm^2^) and G2/M score illustrates phenotype–program coupling; (C) Density contours summarize how predicted identities occupy morpho-transcriptomic space. STAMP: single-cell transcriptomics analysis and multimodal profiling.

While marker genes are sufficient for confirming identity in the STAMP MIX cell line dataset, biological insight often requires moving beyond single genes towards functional gene programs that represent coordinated cellular states. We therefore applied gene module scoring for canonical lineage/state programs, including androgen response, estrogen response, HER2 signaling, and cell-cycle G2/M; followed by visualization on the shared scANVI/UMAP embedding (Figure 3A).

This program-level view recapitulates the expected identity structure (e.g., hormone response and HER2-associated activity correspond to their respective cell lines) while also revealing that the proliferative state is not restricted to a single transcriptional cluster. Instead, the G2/M signal spans the MIX manifold, consistent with the cell cycle behaving as a continuous state axis that intersects otherwise stable identities.

A unique feature of STAMP is that these molecular inferences can be tested against the co-measured phenotype. We refer to this exploratory framework as morpho-transcriptomics: explicitly assessing whether transcriptionally inferred states covary with image-derived morphology. As a proof-of-concept, we asked whether proliferative state correlates with cell size, reasoning that progression through S/G2/M is frequently accompanied by increases in cellular mass and altered morphology. We regressed the G2/M module score against segmented cell area (μm^2^) within MIX and observed detectable associations that illustrate morpho-transcriptomic coupling as a concept (Figure 3B). Given the very large number of single-cell observations, the key interpretive emphasis is on effect size and direction (slope) rather than on *p*-values alone. The modest effect sizes observed (*r* = −0.03 for LNCaP, *r* = −0.18 for MCF7, *r* = 0.19 for SKBR3) and the variation in direction across cell lines indicate that morpho-transcriptomic coupling is most useful as an exploratory proof-of-concept rather than strong validation of transcriptional programs.

The joint distribution of cell area and G2/M score exhibited a characteristic “fan-shaped” pattern: small cells were enriched for low G2/M scores, whereas high G2/M scores preferentially occurred among larger cells, consistent with a size-associated proliferative phenotype (Figure 3B). Stratifying by scANVI-predicted identity further indicated that morpho-transcriptomic coupling can be identity-dependent, with distinct trends across LNCaP, MCF7, and SKBR3 when examined separately (Figure 3B). Finally, density contour visualization provided a compact summary of how each identity occupies a characteristic region of morpho-transcriptomic space (Figure 3C), showing both overlap (shared low-cycling/medium-size regimes) and separation (identity-enriched niches at extremes of size and proliferation).

Together, these analyses illustrate how STAMP enables a transition from “marker-confirmed identity” to mechanistically interpretable state models anchored by co-measured morphology and traceable back to the original images.

### 2.9 Limitations and the “spatial” caveat

In standard STAMP, cells are dissociated before imaging, so the recorded coordinates reflect where cells land on the capture surface rather than their original tissue anatomy. In some implementations, cells may also be cultured or adhered on-slide prior to stamping/readout, but the same caveat applies: coordinates are defined by the capture surface rather than intact-tissue geography. STAMP therefore preserves rich cell-intrinsic spatial information (morphology, polarization, marker localization) but generally does not retain native tissue geography; for anatomy-driven questions, intact-section spatial methods are more appropriate^[2,4]^.

A second limitation is depth/breadth: STAMP is typically panel-based (targeted genes) and does not match whole-transcriptome scRNA-seq for discovery of unexpected pathways^[1]^. This is partly mitigated by a customizable, iterative panel design, in which markers and programs can be expanded/refined across experiments to match the biological question. In addition, emerging higher-content offerings (e.g., whole-transcriptome-style panels for imaging platforms) are beginning to narrow this gap while retaining image linkage.

In spite of these tradeoffs, dissociation enables scalability and experimental flexibility; STAMP can be used as a controlled arena for engineered mixtures, perturbations, and replicates, supporting high-throughput downstream Python analyses of composition, programs, and morpho-transcriptomic coupling.

### 2.10 Where the field is headed

As STAMP-like assays produce larger paired image-expression datasets, bottlenecks increasingly shift from measurement to integration^[28]^. A practical challenge is reliable alignment of segmentation, morphology, and molecule calls into a stable per-cell representation that can be re-queried and re-visualized. Methods for denoising sparse targeted panels, learning latent representations robust to batch and FOV structure^[29]^, and interpreting neighborhoods in dissociated arrays will likely determine how broadly STAMP scales across labs and instruments.

A promising tactic is to treat STAMP as training data for predictive models that map from cell appearance to molecular state. Because each observation includes paired images and expression profiles, STAMP supports supervised and self-supervised learning to predict gene programs from morphology or marker localization, with applications in assay triage, hypothesis generation, and potentially “virtual profiling” workflows where microscopy-guided predictions augment sparse molecular readouts. More broadly, STAMP’s image-registered coordinate system can serve as a scaffold for integrating additional modalities (e.g., multiomic readouts, proteomic panels, or sequential assays on the same slide), enabling cross-platform alignment and joint analysis within a common per-cell reference frame.

## 3. Conclusion

From an analytical viewpoint, STAMP is best viewed as a computational bridge between morphology and molecular profiles. Cell-level metadata (coordinates) generated computationally from STAMP are tightly linked to the source image. Image-aware QC and filtering applied upstream help to interpret the scRNA-seq–inspired inference modules. By linking microscopy evidence to every cell-level molecular measurement, STAMP permits scalable multimodal analyses in which phenotype and genotype can be validated against each other rather than inferred in isolation.

Practically, this pairing is uniquely strong for analyses that require phenotype-grounding: for example, testing whether stress/dissociation-response programs concentrate in morphologically damaged cells, distinguishing true cycling from large quiescent cells using joint size and G2/M scores, and validating inferred epithelial-mesenchymal transition (EMT)-like states against measurable shape and polarization.

## Supplementary Material

Supplementary Material

The supplementary material for this article is available at: Supplementary materials.

## Figures and Tables

**Table 1.** Comparison of measurement modalities and the “spatial” information they retain.

Modality (representative examples)	Sample context	Molecular readout	Spatial/phenotypic information retained	Key strengths and limitations
Droplet-based scRNA-seq, e.g., 10x Chromium, Drop-seq^[1]^	Cells or nuclei dissociated from tissue; native spatial organization is not retained	Whole-transcriptome RNA profiling; RNA only in standard workflows	No native tissue geography, intercellular context, or cell morphology is retained	Highest gene coverage and throughput; extensive analytical ecosystem; enables robust cell-state discovery. Dissociation can alter expression profiles, and native spatial context, cell-cell relationships, and morphology are inaccessible
Spot-based spatial transcriptomics, e.g., Visium ^[3]^, Slide-seq ^[6]^, Slide-seqV2 ^[7]^, Visium HD	Intact tissue sections placed on spatially barcoded capture arrays; native tissue architecture is preserved	Whole-transcriptome RNA profiling on most platforms	Native tissue geography is retained; effective resolution ranges from multicellular spots in standard Visium, approximately 55 μm, to near-cellular features in Slide-seqV2, approximately 10 μm, and subcellular-scale 2 μm bins in Visium HD. Histology provides morphological context, but direct per-cell morphology-transcriptome linkage requires segmentation, cell assignment, or deconvolution	Broad gene coverage with spatial tissue context; supported fresh-frozen and FFPE workflows; mature computational ecosystem. Standard-resolution spots often contain multiple cells, requiring deconvolution for cell-type interpretation; Visium HD improves spatial granularity but still requires computational aggregation or segmentation for robust cell-level analyses
In situ imaging-based spatial transcriptomics — targeted panel, e.g., MERSCOPE/MERFISH ^[8]^, CosMx ^[9]^, Xenium ^[10]^, seqFISH+ ^[11]^	Intact tissue sections; cells are profiled in situ in their native tissue context	Targeted RNA panels, typically hundreds to thousands of genes; RNA ± protein on select platforms; subcellular transcript localization possible	Native tissue geography is retained with single-cell and often subcellular transcript localization. Cell morphology can be captured through DAPI, immunofluorescence, or other imaging channels combined with segmentation	Rich spatial context at single-cell resolution; subcellular detail; RNA ± protein measurement on select platforms. Panel-based design limits transcriptome breadth compared with whole-transcriptome approaches; segmentation quality strongly affects data quality; throughput and panel size vary by platform
Dissociation-based image-linked transcriptomics, e.g., STAMP ^[5]^	Cells or nuclei are dissociated from tissue and immobilized on imaging slides; native tissue geography is not retained	Targeted RNA panel ± protein using cyclic imaging-based readouts	Cell-intrinsic morphology, including area, shape, and marker localization, is retained per cell; slide coordinates are measured but are not equivalent to native tissue geography	Scalable and compatible with dissociated samples; morphology is directly quantified per cell; standard single-cell analysis frameworks can be applied. Native tissue context is sacrificed by dissociation, and panel-based scope limits transcriptome breadth compared with whole-transcriptome methods

scRNA-seq: single-cell RNA sequencing; STAMP: single-cell transcriptomics analysis and multimodal profiling; FFPE: formalin-fixed paraffin-embedded; MERFISH: multiplexed error-robust fluorescence in situ hybridization; HD: high definition; seqFISH+: sequential fluorescence in situ hybridization plus; DAPI: 4′,6-diamidino-2-phenylindole.

**Table 2.** Methods used in this mini review, STAMP downstream workflow and their outputs.

Tool or method	Purpose in STAMP workflow	Key output
Scanpy	Quality control, normalization, feature scaling, neighborhood graph construction, clustering, differential expression, and visualization	Processed AnnData object with per-cell metadata in *obs*, embeddings in *obsm*, UMAP coordinates, cluster labels, and marker-gene rankings from *rank_genes_groups*
scVI	Batch-aware latent modeling, integration across samples or experimental batches, and optional expression denoising	Batch-corrected latent embedding, typically stored in *obsm[“X_scVI”]* or *obsm[“X_scvi”]*; normalized or denoised expression estimates when explicitly computed
scANVI	Semi-supervised cell-type annotation or label transfer using labeled reference cells and unlabeled query cells	Predicted cell labels and posterior probabilities, for example *obs[“scanvi_pred”]* and *obs[“scanvi_maxprob”]*
Module scoring, e.g., *scanpy.tl.score_genes*	Program, pathway, or cell-state scoring using genes represented in the targeted panel	Per-cell signature scores stored in *obs*; interpretation depends on the number and specificity of panel genes available for each signature
Morphology features from segmentation and regionprops	Quantification of cell-intrinsic phenotype variables for quality control, annotation support, and morpho-transcriptomic analysis	Per-cell features such as area, eccentricity, aspect ratio, nuclear or cellular intensity, marker localization, and texture metrics stored in *obs*
Regression, correlation, or stratified analysis	Testing associations between morphology, marker intensity, transcriptomic programs, cell states, perturbations, or sample groups	Effect sizes, confidence intervals, statistical significance, trends across strata, and diagnostic plots

STAMP: single-cell transcriptomics analysis and multimodal profiling; UMAP: Uniform Manifold Approximation and Projection; scVI: single-cell variational inference; scANVI: single-cell ANnotation using variational inference.

## Data Availability

All code used to download the STAMP datasets from public repositories, perform the downstream analyses, and generate all panels in Figure 2 and Figure 3 is publicly available at: https://github.com/surPoudel/STAMP_data_analysis_python.
