# Supplementary material for: Democratizing the spatial view: STAMP technology from an analytical perspective
Source: EXO. Author manuscript; Available in PMC 2026 Jul 21. (PMC13384225; doi:10.70401/EXO.2026.0012)
Supplement: Supplementary Material [file NIHMS2185337-supplement-Supplementary_Material.zip › Supplementary_figures/Figure_S1.pdf]

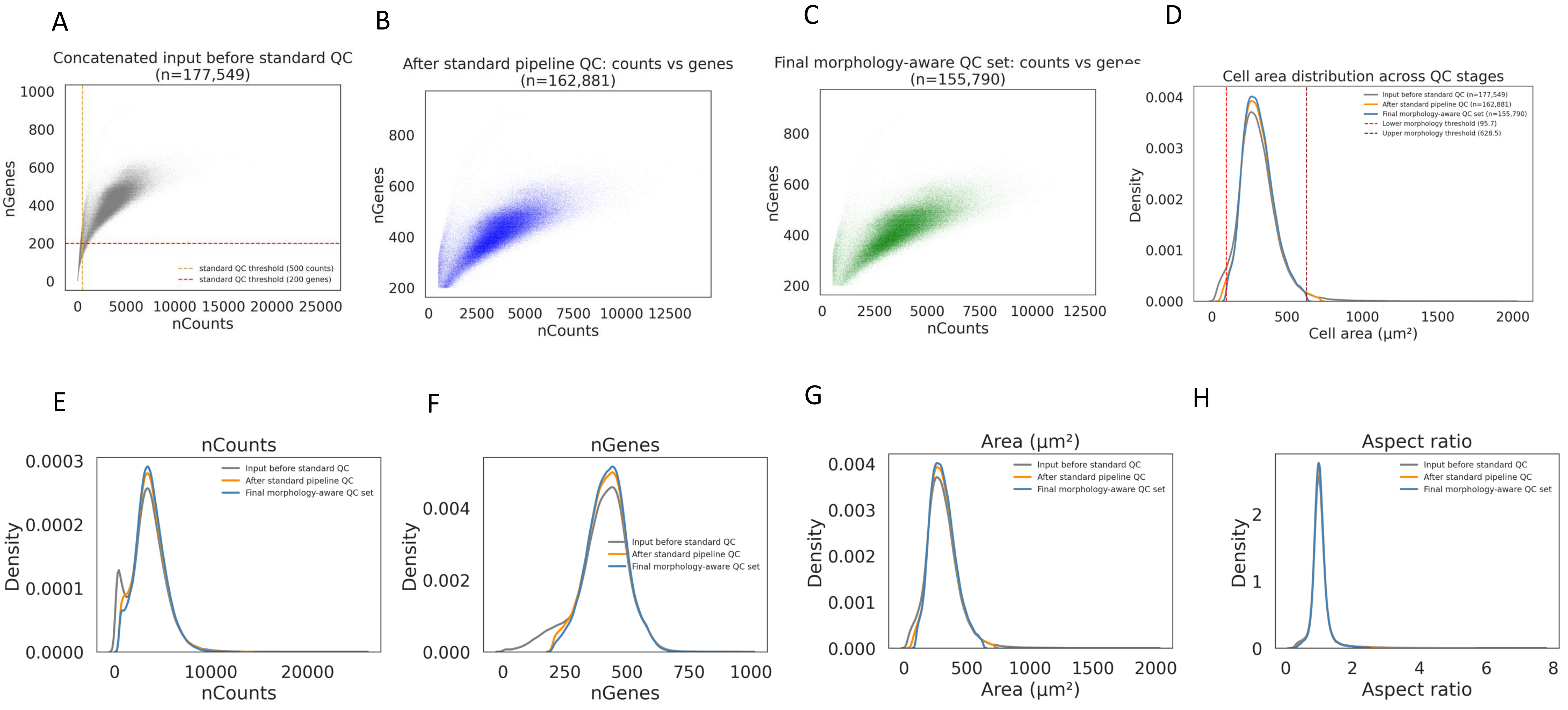

St. Jude - Confidential
