# Supplementary material for: Democratizing the spatial view: STAMP technology from an analytical perspective
Source: EXO. Author manuscript; Available in PMC 2026 Jul 21. (PMC13384225; doi:10.70401/EXO.2026.0012)
Supplement: Supplementary Material [file NIHMS2185337-supplement-Supplementary_Material.zip › Supplementary_figures/Figure_S2.pdf]

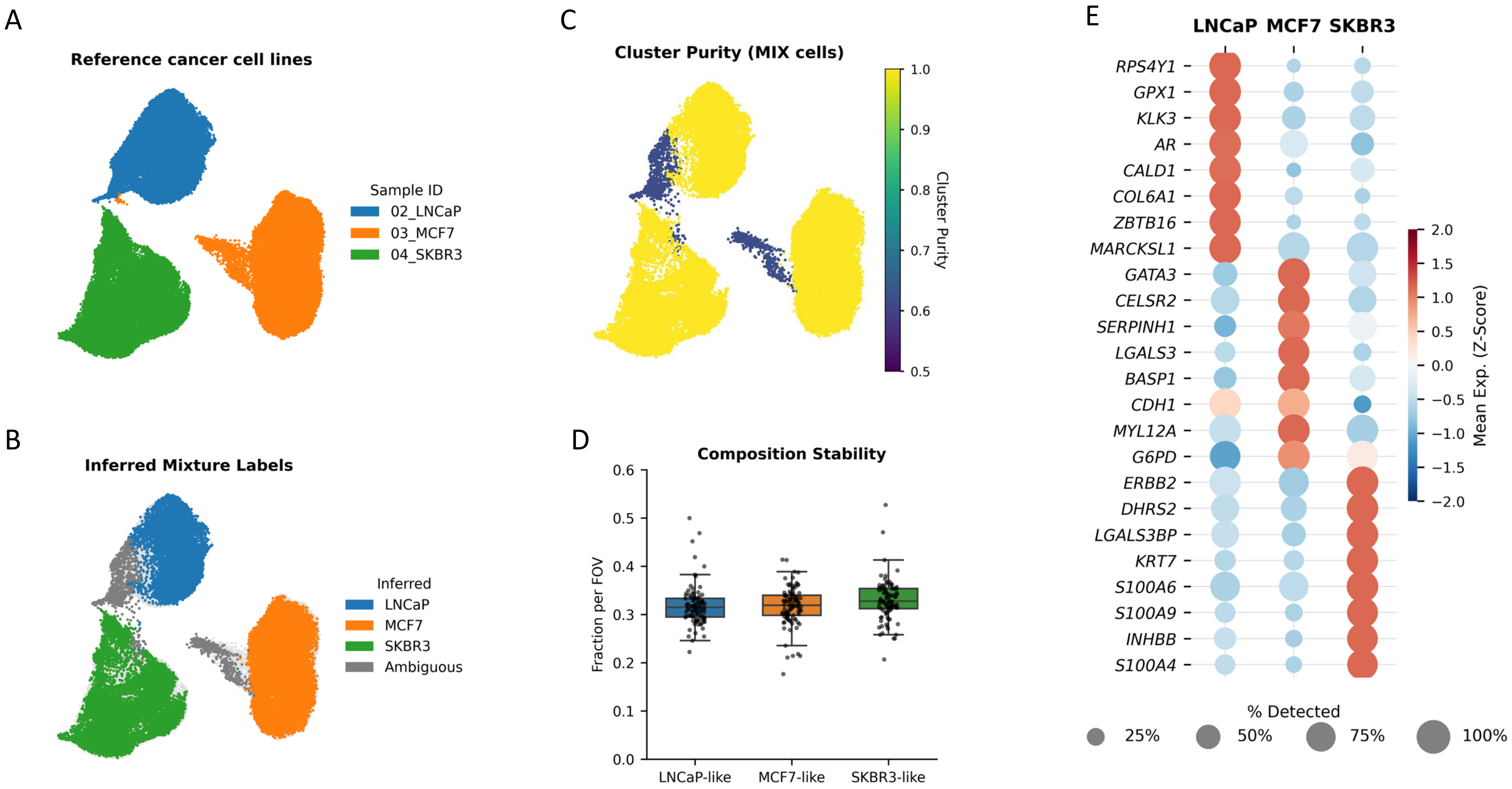
A
Reference cancer cell lines
Sample ID
02_LNCaP
03_MCF7
04_SKBR3
B
Inferred Mixture Labels
Inferred
LNCaP
MCF7
SKBR3
Ambiguous
C
Cluster Purity (MIX cells)
Cluster Purity
1.0
0.9
0.8
0.7
0.6
0.5
D
Composition Stability
Fraction per FOV
0.6
0.5
0.4
0.3
0.2
0.1
0.0
LNCaP-like
MCF7-like
SKBR3-like
E
LNCaP
MCF7
SKBR3
RPS4Y1
GPX1
KLK3
AR
CALD1
COL6A1
ZBTB16
MARCKSL1
GATA3
CELSR2
SERPINH1
LGALS3
BASP1
CDH1
MYL12A
G6PD
ERBB2
DHRS2
LGALS3BP
KRT7
S100A6
S100A9
INHBB
S100A4
Mean Exp. (Z-Score)
2.0
1.5
1.0
0.5
0.0
−0.5
−1.0
−1.5
−2.0
% Detected
25%
50%
75%
100%
